# A new rapid lipoarabinomannan urine assay for tuberculosis: a two-centre diagnostic accuracy evaluation in outpatients with and without HIV

**DOI:** 10.21203/rs.3.rs-5386988/v2

**Published:** 2025-02-18

**Authors:** Beston Hamasur, Anna O. Okunola, James Sserubiri, Welile V. Nwamba, Shima M. Abdulgader, Lech Ignatowich, Omid Rasool, Rose Nabatanzi, Sara Puertas Lorente, David Giraldos, Moses Joloba, Robin M. Warren, Willy Ssengooba, Grant Theron

**Affiliations:** 1 Biopromic AB, Solna, Sweden.; 2 Department of Microbiology, Tumor and Cell Biology, Karolinska Institute.; 3 DSI-NRF Centre of Excellence for Biomedical Tuberculosis Research; South African Medical Research Council Centre for Tuberculosis Research; Division of Molecular Biology and Human Genetics, Faculty of Medicine and Health Sciences, Stellenbosch University, Cape Town, South Africa.; 4 Department of Medical Microbiology, Makerere University, Kampala, Uganda.; 5 Turku Bioscience Centre, University of Turku and Åbo Akademi University, 20520 Turku, Finland.; 6 Department of Immunology and Molecular Biology, Makerere University College of Health Sciences, Kampala, Uganda; 7 Operon S.A. Spain.; 8 Makerere University Lung Institute (MLI), Kampala, Uganda.

**Keywords:** tuberculosis, HIV, diagnosis, urine, lipoarabinomannan

## Abstract

**Introduction:**

Non-sputum tests for people attending primary care with symptoms of tuberculosis (TB) are a global priority.

**Methods:**

We performed a cross-sectional two-centre diagnostic accuracy study of a next-generation urine lipoarabinomannan assay (Biopromic TB LAM, BP-LAM) and the lateral flow Determine TB LAM Ag assay (LF-LAM) on urine from 629 adults with presumptive pulmonary TB (315 with TB) attending primary care in South Africa and Uganda. An extended sputum microbiological reference standard (eMRS) was used and, in South Africa, sputum induction done.

**Results:**

BP-LAM had higher sensitivity than LF-LAM [95% confidence interval (CI) 63% (58, 69) vs. 22% (18, 27)] and similar specificity [93% (90, 96) vs. 89% (85, 92)]. BP-LAM sensitivity did not differ in HIV-positive vs. -negative people nor by CD4 counts. Specificity was diminished in PLHIV [90% (83, 94) vs. 96% (92, 99)]. The design-locked version of BP-LAM had improved specificity than the prototype [93% (90, 96) vs. 85% (80, 88)]. In people with TB who could not expectorate sputum, 67% (55, 79) were BP-LAM-positive; resulting in similar yield to sputum Xpert MTB/RIF Ultra [64% (49, 78)] in a programmatic scenario where sputum induction is unavailable. BP-LAM false-negatives had, vs. true-positives, less severe disease and were more likely to have previous TB. False-positive results were more likely to be from people who cultured only non-tuberculous mycobacteria [19% (4/21) vs. 4% (12/293) for true-negatives].

**Conclusions:**

BP-LAM has higher sensitivity than LF-LAM, including in HIV-negative people, potentially permitting the expansion of urine LAM testing beyond people with advanced immunosuppression.

**Funding:**

The Global Health Technology Fund (GHIT) programs RFP-TRP-2018-001 and RFP-PD-2020-002, EDCTP2 (RIA2020I-3305, CAGE-TB; TMA2020CDF-3209, RADIANT).

## Introduction

In 2023 10.8 million people fell ill with TB^1^. Among these, 1.25 million died, including 161,000 people living with HIV (PLHIV)^1^, for whom TB is the single biggest cause of death. Rapid non-sputum tests, especially those that can be done near point-of-care, are one of the highest priority TB diagnostic needs^2^.

People with TB excrete lipoarabinomannan (LAM), a mycobacterial cell wall glycolipid, in urine^3^. In 2015, World Health Organization (WHO) endorsed the first-generation lateral flow Determine TB LAM Ag assay (LF-LAM; Abbott, Palatine, USA); to date the only commercially available true point-of-care test for TB and the only urine test for TB^4^. However, LF-LAM’s utility decreases as people are less immunosuppressed and have less advanced disease, with sensitivities ranging from ~52% in hospitalised PLHIV to ~29% in PLHIV attending primary care to ~11% in HIV-negative people in primary care^5,6^. Although clinically important, this relatively small group of people in whom the test has the highest sensitivity has, amongst other factors, discouraged widely uptake^4,7^ despite opportunities for increased diagnostic yield^8^. The highly anticipated next-generation Fujifilm SILVAMP TB LAM (FujiLAM; Fujifilm, Tokyo, Japan), showed initial promise (54% overall sensitivity in inpatients and outpatients with HIV) but, after manufacturing challenges, was indefinitely postponed^9^.

Here, we validate the Biopromic TB-LAM assay (BP-LAM; Biopromic, Solna, Sweden), an improved and redesigned version of earlier proof-of-concept work^10,11^. BP-LAM uses a magnetic specimen cleaner method (SpClean; Biopromic) to remove inhibitors and enrich LAM followed by antigen-based immunochromatographic detection within 60 min. BP-LAM has a rabbit anti-LAM IgG monoclonal antibody (Biopromic) as the detection reagent, and an improved chimeric mouse-human anti-LAM IgM/IgG antibody (Biopromic) as the capture reagent that, with SpClean, permit high sensitivity and low costs. Biopromic recently partnered with Operon S.A. (Cuarte de Huerva, Spain) to permit large scale BP-LAM production using a design-locked version (see **Supplementary Text, pg. 2** for more assay technical information).

We evaluated BP-LAM diagnostic accuracy in outpatients self-reporting with presumptive pulmonary TB to clinics in South Africa and Uganda. To inform adoption, we deliberately selected a population in whom testing using the first-generation LAM assay was typically done not. Critically, we used prototype and design-locked versions of BP-LAM and included people not able to naturally expectorate sputum.

## Methods

### Study design and participant eligibility

Adults (≥18 years) with presumptive TB had at least one (PLHIV) or two (HIV-negative) symptoms suggestive of TB (current cough, fever, night sweats, weight loss)^12^ and self-presented to outpatient clinics in Cape Town, South Africa (5 February 2018–21 June 2022) or Kampala, Uganda (14 September 2021–11 March 2022). Participants were excluded if they had TB treatment within the last two months or were of unknown treatment status. This study follows guidance for non-sputum test diagnostic accuracy evaluations^13^ and is reported per the Standards for Reporting of Diagnostic Accuracy Studies^14^. Individual level data is in the spreadsheet in the Supplement.

### Ethics and approvals

The study was approved by the relevant institutional human research ethics committees (South Africa: Stellenbosch University N14/10/136, Western Cape Province WC_2016RP38_944; Uganda: Makerere University SBS-REC SBS-871, Uganda National Council for Science and Technology UNCST HS1527ES). Written informed consent was obtained.

### Data and specimen collection and procedures

Data, including self-reported demographic information and clinical information, were captured using REDCap^15^. HIV testing with counselling was offered. Specimen collection and diagnostic testing are summarised below and in **Supplementary Figure 1**.

#### South Africa:

Participants were required to provide at least two sputa (≥1ml each) in a study (Clinicaltrials.gov NCT03356925) that has, in part, had the performance of sputum^16^ and blood tests reported^17,18^. For the last 44 participants, staff first asked people to expectorate as much sputum as possible per guidelines^19^ and documented this before induction was attempted (we were hence only able to confidently identify sputum scarce people within these 44; prior to this sputum induction was offered but whether people could naturally expectorate first was not recorded). The first sputum was used for a Xpert MTB/RIF Ultra (Ultra) (version 4, Cepheid, Sunnyvale, USA)^20^ and the second 1% NaOH-NALC decontamination with a Mycobacteria Growth Indicator Tube (MGIT) 960 culture (MGIT960; Becton Dickinson Diagnostic Systems, Sparks, USA).

#### Uganda:

Participants were required to provide one sputum specimen (≥3ml). Sputum induction was not offered. Sputum was homogenised by vortexing^21^. The first aliquot was used for Ultra^20^, and the second underwent decontamination and a MGIT960 culture.

### Urine collection

#### South Africa:

Participants were asked for ≥20 ml urine in a 40 ml specimen collection jar. People who could not make urine were offered water to drink and waited. Aliquots were frozen (−20°C) and two 5 ml aliquots shipped for testing at Biopromic.

#### Uganda:

Participants were asked to provide 50 ml of urine and aliquots frozen (−20°C). Two 5 ml aliquots for TB-negatives and three 5 ml aliquots for TB-positives were shipped.

### Participant and urine selection

Participants with urine and a valid (positive or negative) eMRS were tested. South Africa and Uganda each consecutively selected 315 people with TB and 314 randomly selected eMRS-negatives.

### Urine testing

#### BP-LAM procedure:

Urine was thawed at room temperature (RT) and 2 ml transferred using a plastic pipette to a 5 ml polypropylene tube with 100 μl SpClean. After 10 min, the tube was placed in a magnetic rack for 30–60s and cleaned urine transferred to another polypropylene tube. Capture beads (50 μl) with anti-LAM antibodies (Biopromic) were added to concentrate LAM. After mixing and 15 min incubation in a separate non-magnetic rack, the tube was replaced in the magnetic rack for 30–60 s, supernatant removed and discarded, and then, to release captured LAM from beads, 50 μl elution buffer (Biopromic) added and the mixture was incubated back in the separate non-magnetic rack for 5 min. Last, 50 μl neutralisation buffer (Biopromic) was added and the 100 μl mixture immediately applied to the lateral flow assay (LFA) strip.

#### BP-LAM scoring:

After 30 min, LFAs were scored both by eye by two trained operators blinded to all meta-data and each other and using an electronic reader^22^ (Operon S.A.). Sample preparation and product information are in Figure 2 and in this video (https://www.youtube.com/watch?v=XyueJ5ZowAU).

#### LF-LAM:

Testing was done as recommended^23^ using the same aliquot used for BP-LAM with blinding done in a similar manner. For South Africa, LF-LAM was done at the same time as BP-LAM; for Uganda it was done on fresh urine approximately a year prior to LF-LAM). If initial interpretations differed, operators discussed results and reached a consensus.

### Extended microbiological reference standard (eMRS)

A TB case had at least one sputum test positive for *Mycobacterium tuberculosis* complex (MTBC) from Ultra or MGIT960 culture with MPT64 antigen tests (in both countries) and the MTBDR*plus* line probe assay (in South Africa only), used to confirm positive growth. A non-TB was negative by both tests. If one result was missing or unsuccessful, the other was used. Patients were classified as definite TB or non-TB blinded to index test results.

### Statistical analysis

The eMRS was the primary reference standard. An MRS (**Supplement, pg. 2**) was used for secondary analyses. Acid-fast bacilli (AFB)-positive cultures with MTBC-negative growth were designated non-tuberculous mycobacteria (NTMs) and grouped with non-TBs (provided, for eMRS, sputum Ultra-negative). Secondary analyses were done with NTM-positives excluded. Diagnostic yield among all tested (DYT) and diagnostic yield among all those diagnosed (DYD) were calculated^24^. Unless stated, all analyses are head-to-head and BP-LAM diagnostic metrics presented for the design-locked version.

## Results

### Participant characteristics

Urine from 629 people (282 South Africa, 347 Uganda) were evaluated. Ugandans were, compared to South Africans, younger, of higher BMI, less likely to have HIV and previous TB, and had fewer symptoms (Table 1).

### BP-LAM and LF-LAM performance

#### Unsuccessful results, agreement, and quantitative readouts:

No unsuccessful results (not positive or negative) occurred. There was 100% agreement for band lines between readers of each assay. BP-LAM band intensity positively correlated with markers of bacillary load (**Supplementary Figure 2**).

#### Sensitivity:

BP-LAM and LF-LAM had sensitivities of 63% [95% confidence interval (CI) 58, 69] and 22% (18, 27; p<0.001); Table 2 and Figure 3]. For each assay, when sensitivity in PLHIV was compared to within HIV-negative people, non-significant higher point estimates occurred for BP-LAM [67% (58, 76) vs. 62% (55, 69); p=0.394] and LF-LAM [28% (20, 38) vs. 19% (14, 26); p=0.067]. Twenty-four percent (154/629) BP-LAM-positive people were LF-LAM-negative and 6% (38/629) of BP-LAM negative people LF-LAM positive (Figure 4A). The proportion of LF-LAM-positives that were BP-LAM-negative did not differ between PLHIV and HIV-negative people [37% (19/52) vs. 37% (19/52); p>0.999] (Figures 4B–C). BP-LAM positive people were, compared to BP-LAM-negatives, sicker (**Supplementary Table 1**).

#### Specificity:

BP-LAM and LF-LAM had specificities of 93% (90, 96) and 89% (85, 92; p=0.066) (Table 2). In PLHIV, BP-LAM specificity was 90% (83, 94) vs. 85% (77, 91; p=0.271) for LF-LAM. In HIV-negatives, specificities were 96% (92, 99) and 92% (86, 95; p=0.061), respectively. When each assay’s performance was compared in PLHIV vs. HIV-negative people, specificity point estimates were reduced but did not reach significance [BP-LAM: 90% (83, 94) vs. 96% (92, 99), p=0.016; LF-LAM: 85% (77, 91) vs. 92% (86, 95); p=0.069]. Similar findings occurred when analyses excluded Ultra-negative people without a culture and when the MRS was used (**Supplement, pg. 4; Supplementary Table 2**).

#### Performance by country:

BP-LAM specificity and negative predictive value (NPV) were higher in Uganda than South Africa [96 (92, 98) vs. 89 (82, 94); p=0.001; 79 (73, 83) vs. 61 (53, 69); p<0.001]. LF-LAM NPV was also increased [64 (58, 69) vs. 41 (35, 47); p<0.001]. Other metrics and conclusions were similar across countries (**Supplementary Table 3**), however, the proportion of LF-LAM-positives that were BP-LAM-negative was lower in South Africa than Uganda [24% (11/45) vs. 46% (27/59); p=0.025] (**Supplementary Figures 3A-B**).

#### Performance by CD4 count strata:

When sensitivity in people with CD4 counts ≤350 cells/μl was compared to those higher counts, BP-LAM [71% (57, 82) vs. 61% (43, 77); p=0.331] nor LF-LAM sensitivity did not differ [35% (22, 49) vs. 25% (12, 42); p=0.335] (**Supplementary Tables 4–5**).

#### BP-LAM prototype and design-locked versions:

Sensitivity was similar between versions [57% (51, 63) vs. 63% (58, 69); p=0.117] but specificity [85% (80, 88) vs. 93% (90, 96); p<0.001] and PPV [79% (73, 84) vs. 90% (86, 94); p<0.001] of the design-locked version higher. These conclusions were unchanged with the MRS, except for improved specificity and PPV of the design-locked version in HIV-negatives compared to when the eMRS was used (**Supplementary Table 6**).

#### NTM-positive participants:

Five percent (28/595) of cultures were NTM-positive MTBC culture-negative. Of these, 43% (12/28) were eMRS-positive (Ultra-positive). In eMRS-negative NTM-positives, 25% (4/16) were BP-LAM-positive and 19% (3/16; p=0.669) LF-LAM-positive [13% (2/16) positive by both]. In secondary analyses with NTM-positives excluded, small specificity increases occurred (**Supplementary Table 7**).

### Diagnostic yield

#### All participants:

The proportion of BP-LAM-positive people was double that of LF-LAM [35% (220/629) vs. 17% (104/629; p<0.001)]. A greater proportion BP-LAM-positives had a positive sputum test than LF-LAM-positives [90% (199/220) vs. 67% (70/104); p<0.001]. DYTs were 42% (263/629), 48% (299/629), 35% (220/629), and 17% (104/629) for culture, Ultra, BP-LAM, and LF-LAM, respectively (Figure 4, which also shows DYT stratified by HIV status, and **Supplementary Figure 3** which shows DYT stratified by country). Similar conclusions held true for DYD (Table 3).

#### In people with a known sputum scarcity status:

When calculations were restricted to people who had information on whether they required induction, DYTs were 82% (36/44), 86% (38/44), 64% (28/44), and 11% (5/44) for culture, Ultra, BP-LAM, and LF-LAM, respectively (Figure 5A, **Supplementary Table 8**). If we assumed sputum induction were unavailable, sputum test DYT decreased [culture −27% (−46, −8), Ultra −25% (−44, −6)] (Figure 5B, **Supplementary Table 8**) with similar decreases for DYD. In contrast, urine test yield was unaffected and approached that of Ultra and culture. DYDs were 86% (36/42), 90% (38/42), 67% (28/42), and 12% (5/42), respectively (Table 4).

### Factors associated with false BP-LAM results

#### False-positives vs. true-positives:

False-positives were less likely to be male, more likely to be HIV-positive, and less likely to have reported weight loss. BP-LAM quantitative data was lower between false- and true-positives [16 (11–35) vs. 43 (14–691), p<0.001] only in Uganda. (**Supplementary Table 9**).

#### False-negatives vs. true-positives:

Higher CD4 counts among PLHIV, previous TB, and lower burden of disease (longer time-to-positivity of MGIT960 culture, lower Ultra-positive SPC C_T_, and higher *rpoB* C_Tmin_) were associated with false-negativity (and younger age in Uganda). False-negatives had lower BP-LAM quantitative data compared to true-positives [4 (0–9) vs. 48 (14–444), p<0.001; **Supplementary Table 10**].

#### False-negatives vs. true-negatives:

**Figure 1.**
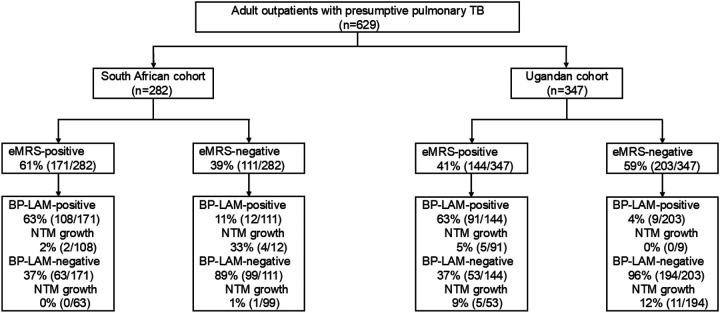
Study profile. Abbreviations: BP-LAM, Biopromic TB LAM assay; eMRS, extended microbiological reference standard; NTM, non-tuberculous mycobacteria positive (and MTBCnegative); TB, tuberculosis.

**Figure 2.**
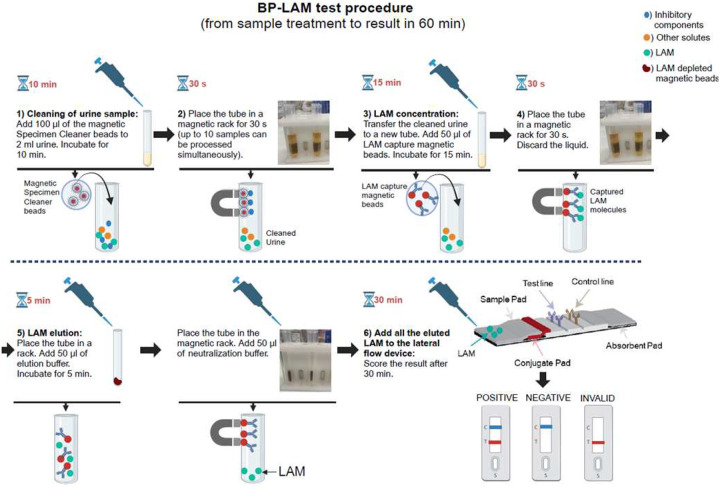
BP-LAM device, procedure, and principle. Abbreviations: BP-LAM, Biopromic TB LAM assay; C, control line; LAM, lipoarabinomannan; T, test line.

**Figure 3.**
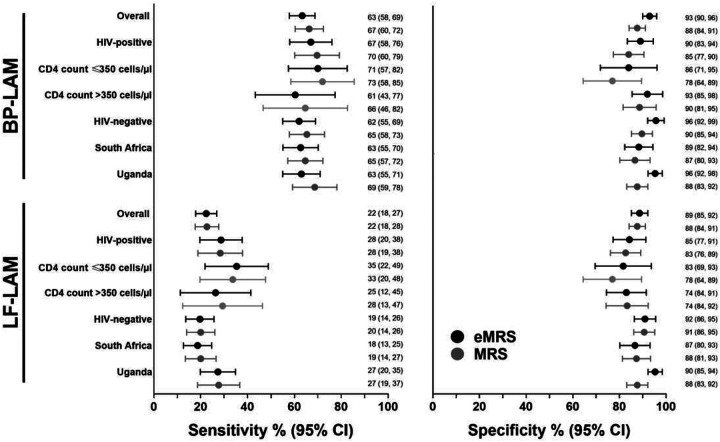
Sensitivity and specificity (95% CIs) of BP-LAM and LF-LAM versus eMRS or MRS across different subgroups. BP-LAM sensitivity was higher than LF-LAM with similar specificity, including after HIV and CD4 count stratification. Abbreviations: BP-LAM, Biopromic TB LAM assay; CI, confidence intervals; eMRS, extended microbiological reference standard; LF-LAM, lateral flow Determine TB LAM Ag assay; MRS, microbiological reference standard.

**Figure 4.**
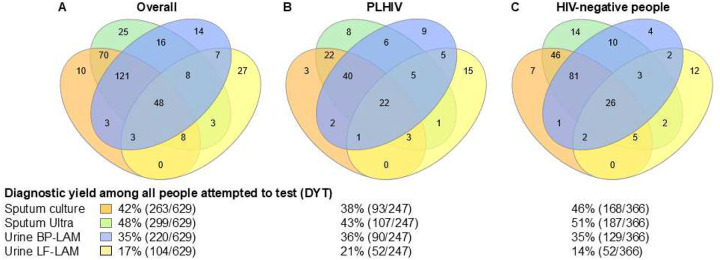
Euler diagrams showing the number of people with a positive test result and DYTs for (A) all people, (B) PLHIV, (C) HIV-negative people, Versions of these Eulers stratified by country is in **Supplementary Figure 3**. Abbreviations: BP-LAM, Biopromic TB LAM assay; LF-LAM, lateral flow Determine TB LAM Ag assay; Ultra, Xpert MTB/RIF Ultra.

**Figure 5.**
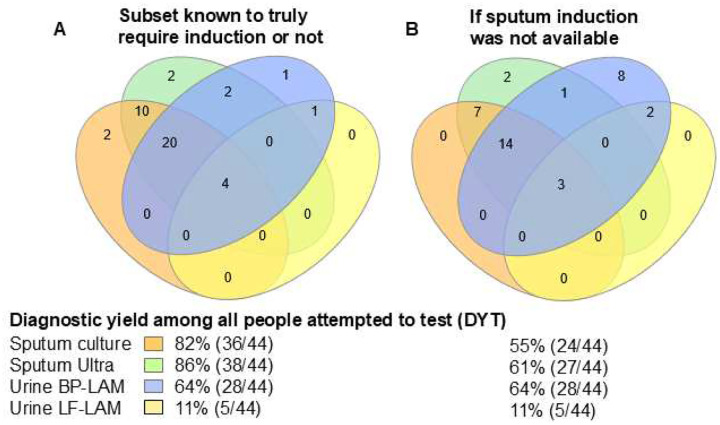
Euler diagrams showing how the number of people with a positive BP-LAM urine result approaches that of sputum tests when sputum induction is unavailable. **(A)** shows, in the subset of people whom sputum scarcity status is known, DYT and **(B)** shows how the numerator for sputum tests decreases when induction is not done. Abbreviations: BP-LAM, Biopromic TB LAM assay; LF-LAM, lateral flow Determine TB LAM Ag assay; Ultra, Xpert MTB/RIF Ultra.

Among eMRS-negatives, 7% (21/314) were BP-LAM false-positive. Of these, 19% (4/21) were NTM-positive (Figure 1). Notably, all four had HIV. One NTM was speciated as *Mycobacterium fortuitum* (**Supplementary Table 11**).

## Discussion

Our key findings are: 1) BP-LAM has higher sensitivity (63% vs. 22%) and similar specificity (~93%) to LF-LAM, 2) BP-LAM had similar DYT to Ultra in a programmatic scenario without sputum induction and diagnosed people with sputum-scarce TB, 3) BP-LAM false-negatives had milder disease than true-positives, suggesting BP-LAM positivity is associated with elevated mortality risk, 4) the BP-LAM design-locked version had comparable sensitivity and improved specificity compared to the prototype, and 5) BP-LAM false-positives were, together with other factors, were more likely to be NTM-positive. Together, these data represent a potential significant step forward for non-sputum testing for TB, especially in outpatients.

BP-LAM had 63% sensitivity (~41% higher than the WHO-endorsed LF-LAM) with comparable specificity (93%). The WHO recently updated the non-sputum rapid test target product profile (TPP) to have minimum sensitivity targets of 65% and 75% (for POC and near-POC, respectively) and >98% specificity^25^. The 95% CIs of our BP-LAM sensitivity estimates did not cross the near-POC target, however, the WHO notes these targets are aspirational, and new tests must be viewed in the context of the existing standard-of-care (LF-LAM) and opportunities for expanding test access (such as permitting people with sputum scarce TB and/or HIV-negativity to be tested).

When BP-LAM sensitivity was compared across HIV statuses, no difference was observed; unlike that described for LF-LAM^26,27^. BP-LAM’s lack (or small) sensitivity decrement in HIV-negative people will likely overcome a major limitation of LF-LAM, whose adoption has lagged due to perceived limited utility despite it being the only TB test with randomised controlled trials showing a mortality benefit^28,29^. A study modelling the impact of different LAM tests on incidence and mortality found LF-LAM could significantly reduce TB deaths among PLHIV with advanced disease, however, such people comprise <1% of total TB cases and deaths^30^. The study further found that if newer more sensitive LAM tests are scaled irrespective of HIV status, population-level impact could be attained with an estimated 18% of TB cases and 30% of TB deaths in high-burden settings like South Africa averted between 2020 and 2035^30^. This modelling estimate was based on a sensitivity estimate of 42% in virally suppressed people, which BP-LAM exceeds.

While BP-LAM had lower yield than sputum Ultra, when we accounted for sputum induction (the absence of which reduces the sputum test yield), Ultra and BP-LAM had similar DYT. This demonstrates the value of BP-LAM (and urine tests in general) and motivates for evaluations of yield in types of people in which sputum scarce TB is most common (e.g., active case finding, children).

Nineteen percent of people without TB who grew an NTM were BP-LAM false-positives (9% for LF-LAM-positive). Potential causes of false-positive LAM results have been discussed extensively and include extrapulmonary TB, reference standard limitations, mixed MTBC-NTM infections with minority MTBC populations, culture bias, and environmental contamination^31^. False-positives require further research with longitudinal follow-up as part of clinical reference standards, especially as our microbiological reference standards depended only on sputum and hence we may be underestimating specificity.

This study has strengths and weaknesses. It consecutively enrolled both HIV-negative people and PLHIV from two epidemiologically different TB-endemic regions in East and South Africa, included the WHO-endorsed LF-LAM as a comparator^12^, and used an eMRS. Further, in South Africa the study included people who could not make sputum and offered them sputum induction. We also compared prototype and designed-locked versions that showed comparable sensitivity, a challenge previously experienced by FujiLAM^9,32^. Our data therefore suggest that BP-LAM may experience less of these challenges, however, this requires prospective monitoring, including stratification of analyses by lot number as production scales. An additional consideration is that, although appropriate for an early evaluation, our study used well-characterised biobanked urine specimens for rapid data generation. Fresh urine may generate different results, however, we assume such studies will likely result in higher BP-LAM sensitivity estimates as LAM in our study might have degraded during storage. Lastly, we noted in Uganda a higher proportion of LF-LAM positive people missed by BP-LAM compared to South Africa. This could be attributable to LF-LAM testing done at a different time-point to BP-LAM (one year earlier in Uganda).

In summary, BP-LAM, a next-generation urine assay, has the potential to enhance rapid TB diagnosis for all people in outpatient clinics with presumptive TB irrespective of HIV status and ability to make sputum. BP-LAM’s diagnostic yield is likely superior to widely-deployed sputum tests like Ultra when applied to populations that include people with sputum scarce TB. BP-LAM performance and effect warrants further prospective evaluation.

## Figures and Tables

**Table 1.** Demographics and clinical characteristics of participants. Data are median (IQR) or % (n/N).

	All participants (N=629)	South Africa (n=282)	Uganda (n=347)	p-value
*Socio-demographic*				
Age, years	34 (19–60)	36 (19–62)	31 (19–56)	**<0.001**
BMI, kg/m^2^	21 (15–34)	20 (15–38)	22 (15–32)	**<0.001**
Sex (male)	60 (376/626)	64 (180/282)	57 (196/344)	0.08
Tobacco smoker (current)	49 (188/383)	51 (145/282)	43 (43/101)	0.13
*Clinical*				
HIV	41 (247/598)	49 (132/272)	34 (115/326)	**0.001**
CD4 count (cells/μl)	377 (24–1108)	343 (18–855)	418 (37–1201)	**<0.001**
Previous TB	28 (173/629)	42 (115/282)	17 (58/347)	**<0.001**
Induction needed to make ≥1 sputum	-	30 (13/44)	Not available	-
*Symptoms*				
Current cough	96 (596/618)	93 (259/280)	99 (337/338)	**<0.001**
≥2 weeks	51 (306/596)	87 (226/259)	24 (80/337)	**<0.001**
Fever	46 (281/616)	30 (84/277)	58 (197/339)	**<0.001**
Weight loss	64 (394/617)	64 (180/280)	64 (214/337)	0.84
Night sweats	57 (356/620)	64 (178/280)	52 (178/340)	**0.01**
Chest pain	59 (366/618)	61 (169/279)	58 (197/339)	0.54
Symptom score (TBscorell)	-	3 (1–7)	Not available	-
*Positive bacteriology*				
Sputum				
Smear microscopy	-	35 (97/274)	Not available	-
eMRS*	50 (315/629)	61 (171/282)	41 (144/347)	**<0.001**
Culture positivity*	44 (263/595)	57 (159/277)	34 (104/318)	**<0.001**
NTM-culture	(5) 28/595	3 (7/277)	7 (21/318)	**0.019**
Urine				
LF-LAM	17 (104/629)	16 (45/282)	17 (59/347)	0.73
If positive, grade	1 (1–3)	1 (1–3)	1 (1–3)	**<0.001**
BP-LAM	35 (220/629)	43 (120/282)	29 (100/347)	**<0.001**
If positive, band intensity from automated reader	5 (0–212)	5 (0–113)	5 (0–252)	**0.28**

**Abbreviations:** BMI, body mass index; BP-LAM, Biopromic TB LAM assay; eMRS: extended microbiological reference standard; LF-LAM, lateral flow Determine TB LAM Ag assay; MRS: microbiological reference standard; TB, tuberculosis.

Missing data: gender, n=3 (Uganda); tobacco smoker (current), n=246 (Uganda); HIV, n=10 (SA) and n=21 (Uganda); sputum induction, n=238 (SA) and n=347 (Uganda); current cough, n=2 (SA) and n=9 (Uganda); fever, n=5 (SA) and n=8 (Uganda); weight loss, n=2 (SA) and n=10 (Uganda); night sweats, n=2 (SA) and n=7 (Uganda); chest pain, n=3 (SA) and n=8 (Uganda); smear microscopy, n=8 (SA); MRS, n=5 (SA) and n=29 (Uganda).

* As described in Methods, an approximate 1:1 ratio of reference standard positives vs. -negatives were selected.

**Table 2.** Diagnostic accuracy of BP-LAM and LF-LAM, stratified by HIV status. BP-LAM sensitivity was higher than LF-LAM with similar specificity, and the sensitivity of both assays were not reduced in HIV-negatives compared to PLHIV. Specificity differed by HIV status for BP-LAM but did not for LF-LAM. Data are %, 95% CI, and n/N.

		All people				HIV-positive				HIV-negative			
		Sensitivity	Specificity	PPV	NPV	Sensitivity	Specificity	PPV	NPV	Sensitivity	Specificity	PPV	NPV
BP-LAM	eMRS	63	93	90	72	67	90	84	76	62	96	95	69
		(58, 69)	(90, 96)	(86, 94)	(67, 76)	(58, 76)	(83, 94)	(75, 91)	(69, 83)	(55, 69)	(92, 99)	(90, 98)	(62, 75)
		199/315	293/314	199/220	293/409	76/113	120/134	76/90	120/157	123/197	163/169	123/129	163/237
										p=0.394*	**p=0.016** *	**p=0.006** *	p=0.203*
	MRS	67	88	81	77	70	85	75	81	65	90	87	74
		(60, 72)	(84, 91)	(76, 86)	(72, 81)	(60, 79)	(77, 90)	(64, 83)	(74, 87)	(58, 73)	(85, 94)	(79, 92)	(67, 79)
		175/263	292/332	175/215	292/380	65/93	120/142	65/87	120/148	110/168	161/178	110/127	161/219
										p=0.467*	p=0.106*	p=0.192*	p=0.093*
		p=0.383^‡^	**p=0.020** ^‡^	**p=0.007** ^‡^	p=0.092^‡^	p=0.685^‡^	p=0.337^‡^	p=0.208^‡^	p=0.342^‡^	p=0.589^‡^	**p=0.027** ^‡^	**p=0.015** ^‡^	p=0.262^‡^
LF-LAM	eMRS	22	89	67	53	28	85	62	58	19	92	73	49
		(18, 27)	(85, 92)	(57, 76)	(49, 58)	(20, 38)	(77, 91)	(47, 75)	(50, 65)	(14, 26)	(86, 95)	(59, 84)	(44, 55)
		70/315	280/314	70/104	280/525	32/113	114/134	32/52	110/191	38/197	155/169	38/52	155/314
		**p<0.001** ^†^	p=0.066^†^	**p<0.001** ^†^	**p<0.001** ^†^	**p<0.001** ^†^	p=0.271^†^	**p=0.002** ^†^	**p=0.001** ^†^	p=0.067*	p=0.069*	p=0.283*	**p=0.046** *
										**p<0.001** ^†^	p=0.061^†^	**p<0.001** ^†^	**p<0.001** ^†^
	MRS	22	88	60	59	28	83	52	63	20	91	67	55
		(18, 28)	(84, 91)	(49, 69)	(54, 63)	(19, 38)	(76, 89)	(37, 66)	(56, 70)	(14, 26)	(86, 95)	(52, 80)	(49, 60)
		59/263	292/332	59/99	292/496	26/93	118/142	26/50	114/181	33/168	162/178	33/49	162/297
		**p<0.001** ^†^	p>0.999^†^	**p<0.001** ^†^	**p<0.001** ^†^	**p<0.001** ^†^	p=0.747^†^	**p=0.007** ^†^	**p=0.001** ^†^	p=0.124*	**p=0.029** *	p=0.120*	**p=0.046** *
										**p<0.001** ^†^	p=0.855^†^	**p=0.003** ^†^	**p<0.001** ^†^
		p=0.968^‡^	p=0.626^‡^	p=0.254^‡^	p=0.075^‡^	p=0.917^‡^	p=0.654^‡^	p=0.341^‡^	p=0.288^‡^	p=0.861^‡^	p=0.815^‡^	p=0.669^‡^	p=0.200^‡^

Within row p-values: *Comparisons by HIV status using the same reference standard.

Within column p-values: ^†^BP-LAM vs. LF-LAM using the same reference standard; ^‡^For each index test comparing eMRS to MRS.

**Abbreviations:** BP-LAM, Biopromic TB LAM assay; CI, confidence interval; eMRS, extended microbiological reference standard; LF-LAM, lateral flow Determine TB LAM Ag assay; MRS, Microbiological reference standard; NPV, negative predictive value; PPV, positive predictive value.

**Table 3.** Diagnostic yield among all people diagnosed (DYD), stratified by HIV status and country. BP-LAM had higher yield than LF-LAM. There was no difference in yield between BP-LAM compared to culture, however, there was within PLHIV and Ugandans. Ultra had higher yield than other tests, irrespective of subgroup. Data are %, 95% CI, and n/N.

	All	HIV-positive	HIV-negative	South Africa	Uganda
*Sputum*					
Culture	72 (68, 77) 263/363	65 (58, 73) 93/142	78 (73, 84) 168/215	83 (78, 88) 159/192	61 (54, 68) 104/171
	^†^ **p=0.001**	^†^p=0.710	^†^ **p<0.001**	^†^ **p<0.001**	^†^p=0.659
Ultra	82 (78, 86) 299/363	75 (68, 82) 107/142	87 (83, 92) 187/215	85 (80, 90) 163/192	80 (74, 86) 136/171
	^‡^ **p<0.001**	^‡^ **p=0.029**	^‡^ **p<0.001**	^‡^ **p<0.001**	^‡^ **p<0.001**
*Urine*					
BP-LAM	61 (56, 66) 220/363	63 (56, 71) 90/142	60 (54, 67) 129/215	63 (56, 69) 120/192	58 (51, 66) 100/171
LF-LAM	29 (24, 33) 104/363	37 (29, 45) 52/142	24 (19, 30) 52/215	23 (17, 29) 45/192	35 (27, 42) 59/171
	* **p<0.001**	* **p<0.001**	* **p<0.001**	* **p<0.001**	* **p<0.001**

Within column p-values for BP-LAM compared to ^†^culture, ^‡^Ultra, and *LF-LAM.

**Abbreviations:** BP-LAM, Biopromic TB LAM assay; CI, confidence interval; LF-LAM, lateral flow Determine TB LAM Ag assay; MRS: microbiological reference standard; TB, tuberculosis; Ultra, Xpert MTB/RIF Ultra.

**Table 4.** Diagnostic yield among all people diagnosed (DYD), stratified by ability to expectorate sputum and in a scenario where induction was unavailable, where BP-LAM had a yield approaching that of culture and Ultra. Data are %, 95% CI, and n/N.

	All	Expectorator	Required induction	If sputum induction was not available
*Sputum*				
Culture	86	83	92	57
	(75, 96)	69, 96)	(77, 99)	(42, 72)
	36/42	24/29	12/13	24/42
			^X^p=0.414	
	^†^ **p=0.040**	^†^p=0.220	^†^p=0.063	^†^p=0.369
Ultra	90	93	85	64
	(81, 99)	(83, 99)	(65, 99)	(49, 78)
	38/42	27/29	11/13	27/42
			^X^p=0.386	
	^‡^ **p=0.008**	^‡^ **p=0.019**	^‡^p=0.185	^‡^p=0.818
*Urine*				
BP-LAM	67	69	62	67
	(55, 79)	(57, 80)	(40, 82)	(55, 79)
	28/42	20/29	8/13	28/42
			^X^p=0.637	
LF-LAM	12	14	8	12
	(3, 26)	(4, 34)	(0, 43)	(3, 26)
	5/42	4/29	1/13	5/42
			^X^p=0.572	
	* **p<0.001**	* **p<0.001**	* **p=0.004**	* **p<0.001**

X Across column p-values: Expectorator vs. required induction

Within column p-values compare BP-LAM to ^†^culture, ^‡^Ultra, and *LF-LAM.

**Abbreviations:** BP-LAM, Biopromic TB LAM assay; CI, confidence interval; LF-LAM, lateral flow Determine TB LAM Ag assay; TB, tuberculosis; Ultra, Xpert MTB/RIF Ultra.

## Data Availability

Data will be made available upon request directly from the corresponding authors and will include de-identified participant data and data dictionaries. The corresponding authors will examine and review data requests. Ethical and legal implications of data sharing will be considered. Data will be shared based on the outcome of the review.
